# The Role of Combination Maintenance with Pemetrexed and Bevacizumab for Advanced Stage Nonsquamous Non-Small Cell Lung Cancer: A Systematic Review and Meta-Analysis

**DOI:** 10.1155/2018/5839081

**Published:** 2018-05-28

**Authors:** Feiyu Shan, Bo Zhang, Leitao Sun, Lufan Xie, Minhe Shen, Shanming Ruan

**Affiliations:** ^1^The First Clinical Medical Institute of Zhejiang Chinese Medical University, Hangzhou 310053, China; ^2^Department of Integrated TCM & Western Medicine, Zhejiang Cancer Hospital, Hangzhou 310022, China; ^3^Department of Medical Oncology, Hangzhou Hospital of Integrated TCM & Western Medicine, Hangzhou 310000, China; ^4^Department of Medical Oncology, The First Affiliated Hospital of Zhejiang Chinese Medical University, Hangzhou 310006, China

## Abstract

**Purpose:**

To evaluate the effect of combination maintenance therapy of pemetrexed plus bevacizumab for patients with advanced non-small cell lung cancer.

**Methods:**

We identified relevant studies by electronic search (Embase, PubMed, Cochrane, and Web of Science from 1 January 1960 to 29 October 2016) and manual search. The primary outcome of interest was progression-free survival (PFS) and secondary end point included overall survival (OS) and toxicities. The data was pooled for quantitative analysis and the final effect size was reported as hazard ratio (HR) for survival outcomes and relative risk (RR) for safety outcomes, both with a random-effects model.

**Results:**

Three randomized controlled trials enrolling 1302 patients with advanced non-small cell lung cancer were included in this meta-analysis. An evident PFS improvement (HR = 0.73, 95% CI = 0.63–0.83, *P* < 0.01) was observed in patients with pemetrexed and bevacizumab combination maintenance therapy compared with single-agent maintenance therapy, yet it did not subsequently lead to a significant improvement in OS (HR = 0.97, 95% CI = 0.84–1.10, *P* = 0.66). Our analysis also showed statistically increased risks for provoking grade 3-4 adverse events in patients managed using pemetrexed plus bevacizumab combination (RR = 1.59, 95% CI = 1.07–2.36, *P* = 0.022).

**Conclusions:**

Pemetrexed plus bevacizumab combination maintenance therapy leads to significant improvement in PFS but not in OS for patients with advanced non-small cell lung cancer, which also increases the risks of grade 3-4 adverse events. Yet, in view of the limitation of existing studies and this meta-analysis, current evidence is not adequate to support routine use of pemetrexed-bevacizumab maintenance.

## 1. Introduction

Until recently, lung cancer remained the leading cause of cancer-related deaths worldwide [1]. At the time of diagnosis, the majority of the lung cancer patients are in advanced stage, which means that there are no curative treatment options. Fewer than 5% of patients with advanced disease stay alive 5 years after diagnosis [2].

With the emergence and development of immune checkpoint inhibitors, for patients who are negative for EGFR mutation, ALK rearrangement, and ROS1 rearrangement and have high programmed death ligand 1 (PD-L1) expression (*⩾*50%), sole use of pembrolizumab has been recommended. But for those who have low PD-L1 expression (<50%), first-line treatment is still considered as a platinum-based doublet chemotherapy [3]. Efforts to improve efficacy have always been focusing on either the selection of agents or the duration of regimens [4–6]. However, these strategies did not yield significant improvements in overall survival (OS) rates and were associated with greater toxicities [6].

Therefore, during the past decades, different research groups have been dedicated to finding alternative strategies to prolong time to disease progression and extend survival. Maintenance therapy, also referred to as consolidation therapy, has been investigated in several large randomized controlled trials and has shown its potential advantages and inconveniences [7, 8]. Bevacizumab and pemetrexed as two effective and well-tolerated agents have been highly recommended as maintenance therapies for patients with advanced nonsquamous non-small cell lung cancer (NSCLC). Recent single-agent maintenance therapy including either bevacizumab or pemetrexed showed that patients with disease controlled after induction therapy may benefit from maintenance therapies before disease progression [7, 9, 10].

Although the combination maintenance regimen of bevacizumab and pemetrexed in 2 large randomized trials indicates improved PFS versus single-agent regimen [11, 12], the risk-benefit ratio of multiagents combination maintenance therapy is still unclear and remains controversial [14]. Hence, there is a pressing need to synthesize this evidence and assess its consistency across trials to achieve a comprehensive summary of the best available evidence for potential benefits of combination maintenance treatments. And it is for this reason that we conduct a meta-analysis of randomized studies to evaluate combination maintenance therapy with bevacizumab plus pemetrexed.

## 2. Methods

### 2.1. Inclusion Criteria

This meta-analysis is reported in line with the Preferred Reporting Items for Systematic Reviews and Meta-Analyses (PRISMA) statement and had been registered at International Prospective Register of Systematic Reviews (number CRD42017062313) [15]. The studies were regarded as eligible for inclusion if they meet all the following criteria: (1) they were RCTs; (2) the study population consisted of patients with histologically or cytologically proven stage IIIB or IV NSCLC; (3) the study contained an intervention group, combination maintenance with pemetrexed plus bevacizumab, and a control group, any other maintenance therapy or no maintenance therapy; (4) they reported at least one of the following outcomes: progression-free survival (PFS), overall survival (OS), and treatment-related toxicities (adverse event grade ≥ 3, AEs).

### 2.2. Search Strategy

We identified relevant studies by searching public databases (Embase, PubMed, Cochrane, and Web of Science from 1 January 1960 to 29 October 2016) and manual searching via reference lists of key articles. The search was restricted to English only. The complete text and MeSH terms searched in PubMed were (Carcinoma, Non-Small-Cell Lung [MeSH Terms] OR Carcinoma, Non Small Cell Lung[Text Word] OR Carcinomas, Non-Small-Cell Lung[Text Word] OR Carcinoma, Non-Small Cell Lung [Text Word] OR Lung Carcinoma, Non-Small-Cell [Text Word] OR Lung Carcinomas, Non-Small-Cell [Text Word] OR Nonsmall Cell Lung Cancer [Text Word] OR Non-Small Cell Lung Cancer [Text Word] OR Non-Small-Cell Lung Carcinoma [Text Word] OR Non Small Cell Lung Carcinoma[Text Word] OR Non-Small-Cell Lung Carcinomas [Text Word]) AND (Bevacizumab [MeSH Terms] OR Avastin [Text word])) AND (Pemetrexed [MeSH Terms] OR Pemetrexed Disodium [Text word] OR Disodium, Pemetrexed [Text word] OR MTA [Text word]))) AND (Randomized Controlled Trial [Publication Type] OR Randomized [Title/Abstract] OR Placebo [Title/Abstract])))).

### 2.3. Study Selection and Data Collection

Two investigators (Leitao Sun and Lufan Xie) performed title and abstract screening independently; among the initial results, those that might meet the inclusion criteria were acquired for further full-text evaluation.

The data extraction was carried out by two independent investigators (Feiyu Shan and Bo Zhang), including general information such as first author's name, year of publication, trial design, number of participants, sex, age, smoking history, ECOG score, and follow-up duration and specific data such as induction chemotherapy regimen, maintenance therapy agents, details of survival, and safety outcomes. The primary outcome of interest was progression-free survival (PFS) and secondary end point included overall survival (OS) and toxicities. The outcome data were pooled as hazard ratio (HR) and relative risk (RR). On account of multiple publications on one trial, the latest evidence was applied.

### 2.4. Risk of Bias Assessment

Two independent investigators (Feiyu Shan and Bo Zhang) performed the quality assessment for studies selected, according to the Cochrane Collaboration's tool for assessing risk of bias. Briefly, the main questions for quality assessment were listed as randomization sequence generation, concealment of allocation, blinding of participants and personnel, blinding of outcome assessment, incomplete outcome data, selective reporting, and other biases. One of three levels categorized as high risk of bias, low risk of bias, or unclear risk of bias was assigned to each item for individual study. Discrepancies were resolved by the third investigator (Shanming Ruan).

### 2.5. Statistical Analysis

The pertinent data was extracted from individual included trials in accordance with the methods described previously [15, 16]. Then the data was pooled for further analysis and the final effect size was reported as hazard ratio (HR) with a random-effects model, where the HR less than 1 betokened an advantage for double-maintenance regimen consisting of bevacizumab and pemetrexed. Results of the meta-analysis were displayed as forest plots. For safety analysis, we calculated the pooled data of relative risk (RR) with a random-effects model. The formal meta-analysis was conducted by STATA (Stata Corporation, College Station, TX; version 12.0).

We assessed the heterogeneity between studies by I^2^ regarding a value greater than 50% as an indicator of moderate-to-high heterogeneity [17]. Egger and Begg tests were used to check for possible publication bias, where values of *P* < 0.05 were defined as significant publication bias. Preplanned sensitivity analysis was conducted for the outcomes of PFS and OS.

### 2.6. Evidence Quality Assessment

The pooled evidence and each outcome were evaluated and classified into one of four categories (high, moderate, low, and very low) of evidence quality referring to GRADE (Grading of Recommendations, Assessment, Development, and Evaluation) [18]. The GRADEpro software (version 3.6.1, Grade Working Group) was used.

## 3. Results

### 3.1. Trial Selection

**Figure 1:**
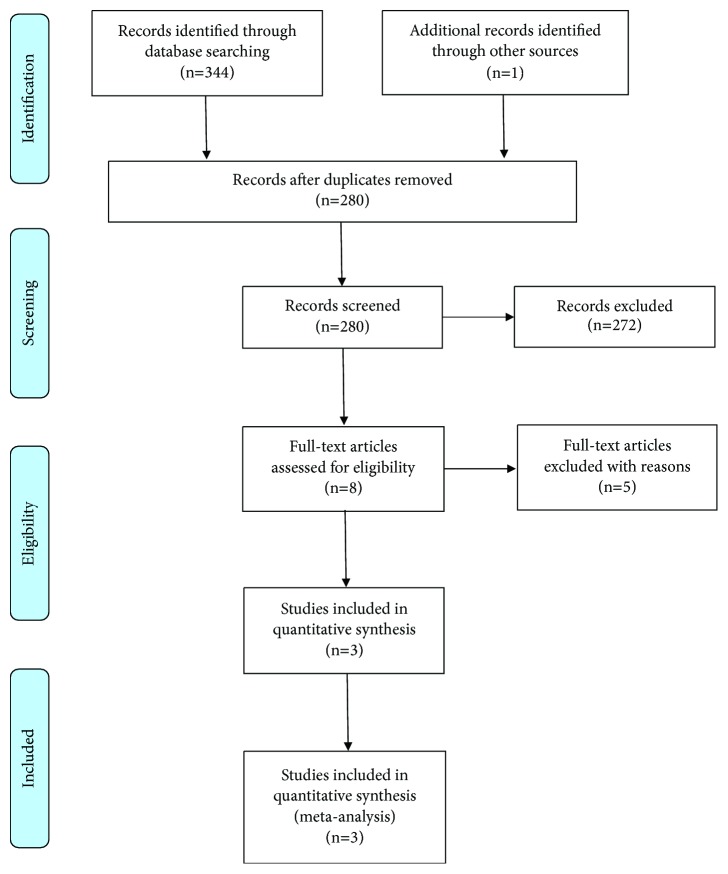
Flowchart showing study selection process. After screening process, only 3 RCT articles met the inclusion criteria and were included in ultimate analysis. RCT = randomized controlled trials.

By the initial search, we identified a total of 345 citations from online database and other sources. After removal of 65 duplicate studies, 280 articles were left for screening. During the further review of the remaining studies, additional 272 articles were excluded for several reasons including being not about NSCLC, review, being not with maintenance therapy, case report, meeting, being not a clinical trial, having other maintenance regimens, and being not a randomized controlled trial. 8 studies were subsequently retrieved for full-text assessment for eligibility, among which 5 publications were excluded for one of the following reasons: review, single-arm study, being not with maintenance therapy, and earlier publication on one trial. In the end, 3 randomized controlled trials about the studies of combination maintenance therapy with pemetrexed and bevacizumab were included [11–13]. The search and selection steps are shown in Figure 1.

### 3.2. Characteristics of Included Studies

3 included randomized controlled trials evaluated 5 maintenance regimens with a total of 1302 patients enrolled. One of the 3 studies (J. Patel et al.) [11] adopted an induction chemotherapy regimen as pemetrexed 500 mg/m2 plus carboplatin AUC 6 plus bevacizumab 15 mg/kg in intervention arm and paclitaxel 200 mg/m2 plus carboplatin AUC 6 plus bevacizumab 15 mg/kg in control arm, both for four 21-day cycles. The maintenance regimen was pemetrexed 500 mg/m2 plus bevacizumab 15 mg/kg and single-agent bevacizumab 15 mg/kg for intervention arm and control arm, respectively. The remaining two trials used same agents in their induction phase for both arms. F. Barlesi et al. [12] used pemetrexed 500 mg/m2 plus Cisplatin 75 mg/m2 plus bevacizumab 7.5 mg/kg as an induction therapy followed by pemetrexed 500 mg/m2 plus bevacizumab 7.5 mg/kg for intervention arm and bevacizumab 7.5 mg/kg alone for control arm. M. Karayama et al. [13] took an induction therapy composed of pemetrexed 500 mg/m2 plus carboplatin AUC 6 plus bevacizumab 15 mg/kg in both groups, while the maintenance therapy was pemetrexed 500 mg/m2 plus bevacizumab 15 mg/kg for intervention arm and pemetrexed 500 mg/m2 for control arm. Two trials reported subgroup analysis according to age, sex, ECOG PS score, and smoking history. Median age (65 years, ranging from 60 to 66), being male (56%), ECOG PS (0, 49%/1-2, 51%), stage (IIIB, 9%/IV, 90%), histology (adenocarcinoma, 83%/other, 17%), smoking history (never, 15%/ever, 85%) are some of the other features of the 3 included trials as presented in Table 1.

### 3.3. Risk of Bias in Individual Study

**Figure 2:**
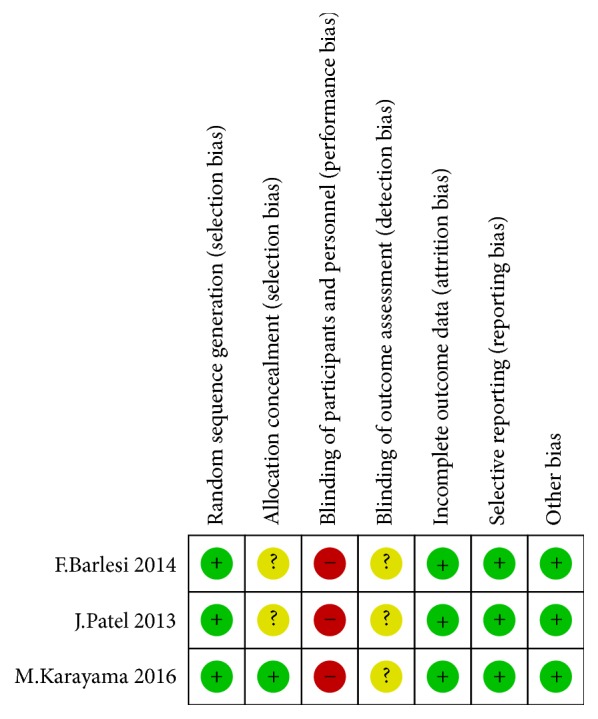
Risk of bias assessment in each item. -: high risk of bias; ?: unclear risk of bias; +: low risk of bias.

**Figure 3:**
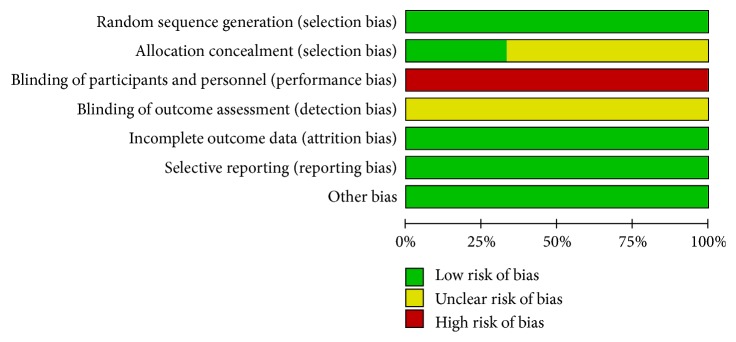
Risk of bias summary.

The assessment of risk of bias items within each of the included studies is listed in Table 2 and Figure 2. All three trials were multicenter with adequate randomization. One of them reported concealment of allocation by central randomization [13]. None used double blind method and the blinding of assessors is not informed in all included trials. All RCTs provided complete outcome data and none reported outcomes selectively. The summary of risk of bias for each item is shown in Figure 3.

### 3.4. PFS Outcomes

**Figure 4:**
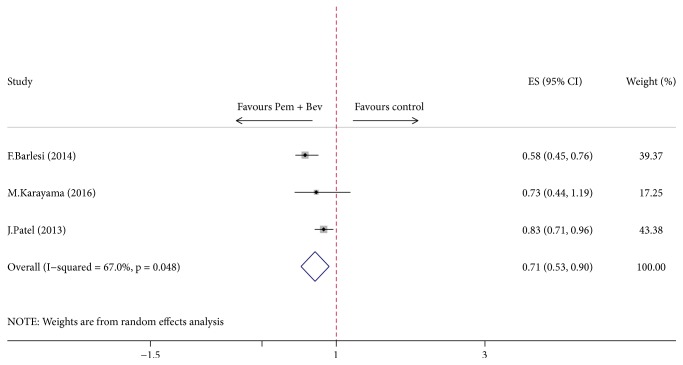
Comparison of progression-free survival between Pem + Bev maintenance and other maintenance regimens. Pem: pemetrexed; Bev: bevacizumab.

An evident PFS improvement (HR = 0.73, 95% CI = 0.63–0.83, *P* < 0.01) was observed in patients with pemetrexed and bevacizumab combination maintenance therapy, which significantly outperformed other single-agent maintenance therapy regimens with either pemetrexed or bevacizumab. However, a moderate degree of heterogeneity existed across the trials (Figure 4). Notably, the overall results were not affected after sequential exclusion of each trial. Considering the serious risk of bias existing within individual study described above and the synthesized result denoting a moderate-to-high heterogeneity across the studies, a low quality of evidence and a weak recommendation were assigned to the pooled evidence of PFS.

### 3.5. OS Outcomes

**Figure 5:**
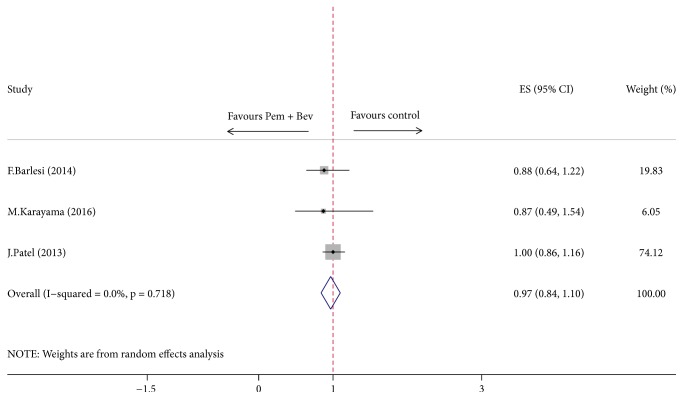
Comparison of overall survival between Pem + Bev maintenance and other maintenance regimens. Pem: pemetrexed; Bev: bevacizumab.

The combination of pemetrexed and bevacizumab as maintenance regimen did not lead to a significant improvement in OS (HR = 0.97, 95% CI = 0.84–1.10, *P* = 0.06) (Figure 5), with no significant heterogeneity across included trials. And the synthesis results remain consistent with original pooled analysis after exclusion of each study seriatim.

### 3.6. Adverse Events

**Figure 6:**
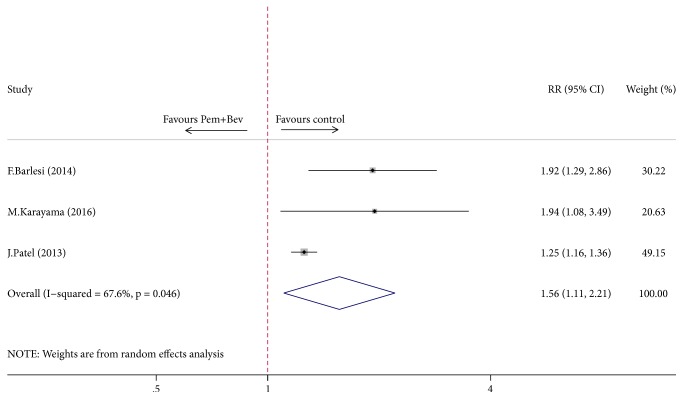
Comparison of overall adverse events between Pem + Bev maintenance and other maintenance regimens. Pem: pemetrexed; Bev: bevacizumab.

A meta-analysis was performed to evaluate the risk of adverse events of grade 3 or above in combination arms and control arms. We found that 74% of patients experienced grade 3-4 adverse events in pemetrexed plus bevacizumab combination arm, while the percentage was 55% in control arm. Comparison between two groups showed statistically increased risks for provoking grade 3-4 adverse events in patients managed using pemetrexed plus bevacizumab combination (RR = 1.56, 95% CI = 1.11–2.21, *P* = 0.012, Figure 6).

**Figure 7:**
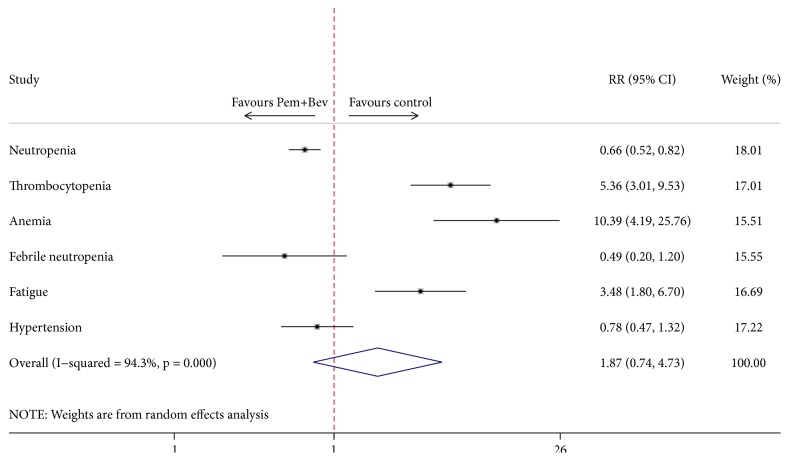
Comparison of specific adverse events between Pem + Bev maintenance and other maintenance regimens. Pem: pemetrexed; Bev: bevacizumab.

Specifically, we observed significantly higher risk of grade 3-4 adverse events for thrombocytopenia (RR = 5.36, 95% CI = 3.01–9.53, *P* < 0.01), anemia (RR = 10.39, 95% CI = 4.19–25.76, *P* < 0.01), and fatigue (RR = 3.48, 95% CI = 1.80–6.70, *P* < 0.01) in combination arm (Figure 7 and Table 3). In contrast, application of the combination regimen of pemetrexed and bevacizumab did not lead to increased risk of neutropenia (RR = 0.66, 95% CI = 0.52–0.82, *P* < 0.01). Although no statistical significance was detected, the analysis also showed lower risks for febrile neutropenia (RR = 0.49, 95% CI = 0.20–1.20, *P* = 0.120) and hypertension (RR = 0.78, 95% CI = 0.47–1.32, *P* = 0.361) (Figure 7 and Table 3).

### 3.7. Subgroup Analysis

**Figure 8:**
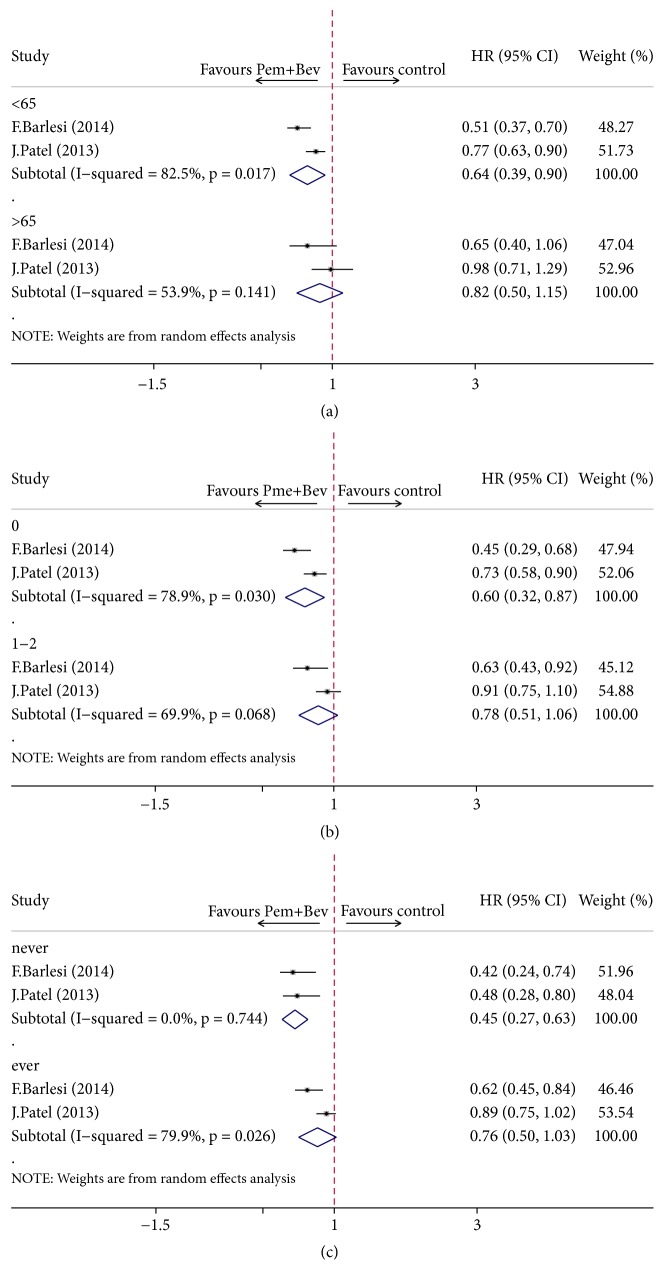
Subgroup analysis of progression-free survival defined by age, ECOG score, and smoking history. (a) Progression-free survival based on age. (b) Progression-free survival based on ECOG score. (c) Progression-free survival based on smoking history. Pem: pemetrexed; Bev: bevacizumab.

**Figure 9:**
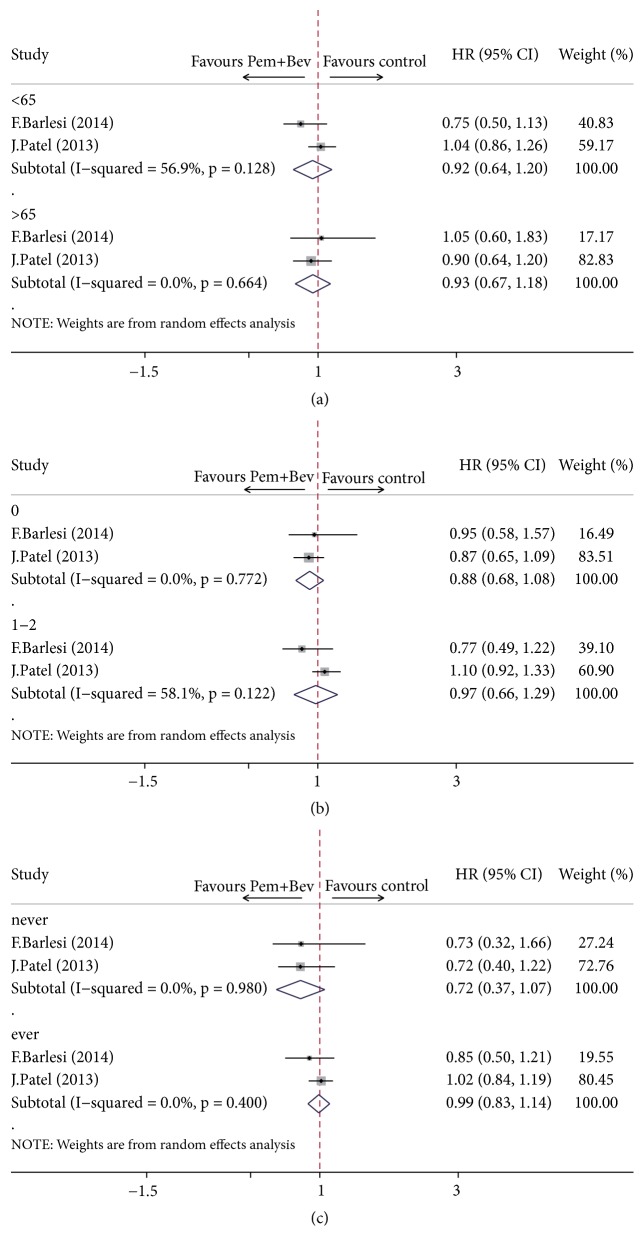
Subgroup analysis of overall survival defined by age, ECOG score, and smoking history. (a) Overall survival based on age. (b) Overall survival based on ECOG score. (c) Overall survival based on smoking history. Pem: pemetrexed; Bev: bevacizumab.

In two of the included randomized trials [11, 12], patients were subdivided according to baseline characteristics such as age, sex, ECOG (Eastern Cooperative Oncology Group) score, smoking history, ethnicity, and histology. We extracted age (< 65 versus ≥ 65 years), ECOG PS (0 versus 1-2), and smoking history (never versus ever), which were owned by both trials collectively, as independent variables and conducted subgroup analysis. As shown in Figure 8, patients managed using the combination strategy appeared to be at an advantage with regard to PFS compared with patients receiving other maintenance regimens when based on subset factors of age, ECOG score, and smoking history. And, remarkably, lower hazard ratios were observed in patients with younger age (< 65, HR = 0.64, 95% CI = 0.39–0.90, *P* < 0.01), better physical status (ECOG score = 0, HR = 0.60, 95% CI = 0.32–0.87, *P* < 0.01), and no smoking history (never smoked, HR = 0.45, 95% CI = 0.27–0.63, *P* < 0.01). As for overall survival, a clear trend for longer OS was observed in patients with age < 65 years (HR = 0.93, 95% CI = 0.69-1.26,* P* = 0.64), ECOG score = 0 (HR = 0.89, 95% CI = 0.69-1.14,* P* = 0.36), or never smoked (HR = 0.72, 95% CI = 0.45-1.17,* P* = 0.18). However, no statistically significant improvement was detected in all three subsets (Figure 9).

### 3.8. Levels of Evidence

**Figure 10:**
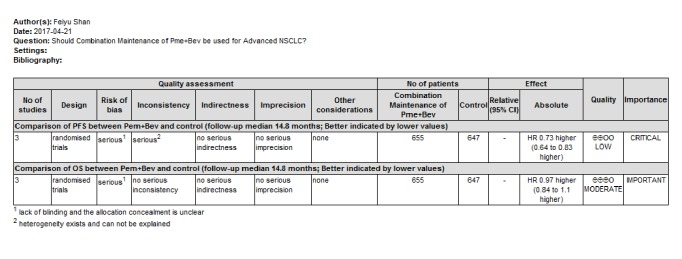
Assessment of evidence of survival outcomes.

In risk of bias domain, due to the lack of double blind method in all trials and unclear risk of detection bias, the PFS and OS outcomes were both initially classified as serious risk of bias. The inconsistency domain was downgraded to serious for heterogeneity that cannot be explained in PFS outcomes. Thus, a low quality was assigned to PFS outcome and moderate quality to OS outcome, respectively. The summary of evidence assessment was shown in Figure 10.

### 3.9. Publication Bias

The results of Begg's test and Egger's test of PFS (P_Begg_ = 1.000, P_Egger_ = 0.618) and OS (P_Begg_ = 1.000,P_Egger_ = 0.286) suggested no publication bias for the present studies, though the limited trials number may reduce the test's efficacy.

## 4. Discussion

### 4.1. Summary of Main Findings

In this meta-analysis reporting the efficacy of pemetrexed and bevacizumab combination regimen as maintenance treatment for advanced NSCLC patients, only three randomized controlled trials met the inclusion criteria. Our results suggest that, compared with single-agent therapy, pemetrexed plus bevacizumab combination as continuous maintenance therapy can yield salient benefits in prolonging PFS. However, there is no significant improvement for OS in the patients receiving regimen of pemetrexed concomitant with bevacizumab in the maintenance setting. Furthermore, it is worth noting that the reduction of risk in survival may be accompanied by increased risks of treatment-related toxicities. These data, to some extent, lend support to pemetrexed and bevacizumab combination therapy as a maintenance strategy that is able to improve the management of advanced non-small cell lung cancer.

### 4.2. Applicability of the Current Evidence

In the management of advanced stage NSCLC, several randomized trials demonstrating statistically significant benefit of PFS and/or OS has revived the notion of accepting maintenance therapy as an appropriate option in making clinical strategy [19–21], although it is not clear whether the benefit is derived from induction phase, maintenance phase, or the integration of them. In a phase III study (ECOG 4599), continuation of bevacizumab beyond six cycles showed 2-month improvement in both median PFS (6.2 versus 4.5 months; HR = 0.66; *P* < 0.01) and median OS (12.3 versus 10.3 months; HR = 0.79; *P* < 0.01) [9], which established the induction of carboplatin plus paclitaxel and bevacizumab followed by bevacizumab continuous maintenance as a standard first-line regimen for advanced NSCLC patients. And pemetrexed, known as multitargeted antifolate, also has been proven with prolonged survival in two double blind, phase III, randomized controlled studies compared with placebo [7, 10]. However, no consensus has been forged regarding the combination usage of pemetrexed and bevacizumab [22]. A single-arm phase II study exploring combination of pemetrexed and bevacizumab in maintenance set have shown encouraging efficacy end point of both PFS (7.8 months, 95% CI = 5.2–11.5) and OS (14.1 months, 95% CI = 10.8–19.6) with acceptable toxicity [23]. And, subsequently, it lays the foundation for the following three phase III combination maintenance trials: AVAPERL, PointBreak, and ECOG 5508, two of which reported significantly improved PFS [11, 12] and results from the third study have not been reported [24]. In this context, the combination of pemetrexed with bevacizumab has been postulated as a potential strategy that may generate benefits in PFS and OS for patients with first-line nonsquamous non-small cell lung cancer [25].

As illustrated in Figure 4, our analysis has yielded a robust finding indicating a beneficial effect on PFS when using pemetrexed and bevacizumab combination maintenance versus either pemetrexed or bevacizumab alone. However, the prolonged PFS failed to translate into a longer OS (Figure 5). The significant improvement in PFS is consistent with the results from two of the randomized trials included in this analysis [11, 12] as well as with another randomized ATLAS study that contained bevacizumab-erlotinib combination [26]. ATLAS reported 1-month improvement in PFS for patients receiving combination maintenance of bevacizumab and erlotinib (4.8 versus 3.7 months; HR = 0.72, *P* < 0.01) but it was not statistically significant in OS (14.4 versus 13.3 months; HR = 0.92; *P* = 0.534) [26]. Meanwhile, although longer median OS was reported in AVAPERL [12] trial in our study with a noteworthy duration of OS exceeding 19 months in pemetrexed plus bevacizumab maintenance arm (19.8 versus 15.9 months), no statistical significance was reached, while PFS was significantly improved (10.2 versus 6.6 months; HR = 0.58; *P* < 0.01). Another trial by Patel et al. [11] powered to detect a statistical difference in OS unfortunately failed to achieve an improved OS (12.6 versus 13.4 months; HR = 1.00; *P* = 0.949), while PFS was statistically superior in combination group (6.0 versus 5.6 months; HR = 0.83; *P* = 0.012), which is possibly due to a high proportion of patients receiving further treatments after the study. As a caveat, the most remarkable feature of PointBreak trial [11] is the different regimen of induction treatment designed as pemetrexed/carboplatin/bevacizumab followed by pemetrexed/bevacizumab (Pem Arm) versus paclitaxel/carboplatin/bevacizumab followed by bevacizumab (Pac Arm), which has made it more difficult to assess the efficacy of maintenance therapy. Yet, when looking only at the 63% of patients who went on to maintenance therapy (the others dropping off because of progression, prohibitive side effects, or other complications), both PFS and OS are longer (by 1.7 and 2.0 months, resp.), suggesting that there may be benefit in taking the two drugs over one for maintenance population. Trend in outcomes similar to PointBreak trial can also be observed in several other phase III randomized trials [26, 27] evaluating the role of pemetrexed and bevacizumab or bevacizumab plus erlotinib in maintenance set. But, notably, subgroup analysis of PFS showed that maintenance of bevacizumab combined with pemetrexed was favored over bevacizumab or pemetrexed alone irrespective of patient age, ECOG score, and smoking history (Figures 8 and 9). And age < 65 year, ECOG score of 0, and no smoking history may be associated with greater survival benefit in patients managed with double maintenance of pemetrexed and bevacizumab. The weight of our evidence, despite lack of high-quality studies and strong recommendation, suggests that pemetrexed and bevacizumab combination has positive effect for patients with advanced NSCLC. And indeed our study shows individual variability in several ways, such as modified dosage of maintenance agent, whether the ITT principle is available, and different components of induction chemotherapy in two arms, which may partly be associated with the heterogeneity between studies.

Prolonged use of pemetrexed combined with bevacizumab for maintenance therapy of NSCLC is burdened by increased risks of treatment-related toxicities, although mostly safe and tolerable. Anemia and thrombocytopenia are two of the prominent adverse events, which account for a large part of incidents causing delay or discontinuation of chemotherapy. Fatigue is another side effect of extended use of pemetrexed and bevacizumab. In this study, either the risks or incidence of grade 3-4 fatigue or anemia or thrombocytopenia is much higher with combination use of pemetrexed and bevacizumab compared with single-agent maintenance, consistent with the current clinical practice experience. Notably, combination maintenance is associated with a much lower risk of neutropenia (RR = 0.66, 95% CI = 0.52–0.82, *P* < 0.01). Febrile neutropenia and hypertension also can be found with a decreased risk of combination regimen, but no statistical significance is detected. Aforementioned results are mainly consistent with the observation of PointBreak trial [11]. And PRONOUNCE study also reported similar results.

### 4.3. Implications of This Review

To our knowledge, no similar comprehensive meta-analysis investigating the benefits and risks of pemetrexed plus bevacizumab maintenance therapy has been done previously. Our study may provide potential implications for clinical practice and health policy. The present findings from our meta-analysis, although based upon only three randomized controlled studies, indicate that pemetrexed plus bevacizumab beyond initial four to six cycles of chemotherapy can significantly improve median PFS and failed to achieve positive progress in OS, while accompanied by potentially increased risks of adverse events. These results suggest that the controversy should shift to whether the benefit in PFS outcomes is of sufficient clinical importance to widely warrant the combination maintenance treatment with pemetrexed and bevacizumab. Considering several additional factors such as patient's preferences, the adverse effects, and cost-benefit ratio of therapy, due caution should be exercised in the decision of using pemetrexed plus bevacizumab combination maintenance. However, the combination maintenance of pemetrexed plus bevacizumab still shows clinically meaningful PFS benefit, especially when administered to advanced non-small cell lung cancer patients with younger age, good performance status, and no smoking history. An ongoing trial ECOG 5508 [24], comparing maintenance with bevacizumab, bevacizumab combined with pemetrexed, and pemetrexed alone, should further elucidate the efficacy and value of combination of pemetrexed plus bevacizumab.

### 4.4. Limitations

Several limitations exist in our study. First, the data extracted is merely from previous publication, whereas original data and individual patient data are unavailable, which make us unable to perform more detailed analysis and obtain more comprehensive results. Second, our analysis is limited by substantial heterogeneity across included trials, which is possibly attributed to the variation in trial design, inclusion and exclusion criteria, and treatment regimen involving induction modalities and agents' dosage. Third, even though most of the included trials were published in journals with high impact factor, open-label design and pharmaceutical industry funding as potential risks of bias still exist. Finally, this meta-analysis is limited by lack of available studies. Thus, these results should be interpreted with caution.

## 5. Conclusion

Our study suggests that the double maintenance of pemetrexed and bevacizumab is associated with significantly prolonged PFS but not OS and is accompanied by increased risks of grade 3-4 adverse events. Given the current limitation of existing studies and this meta-analysis, further studies like ECOG 5508 are expected to report a fundamental strategy and provide a powerful clinical evidence.

## Figures and Tables

**Table 1:** Characteristics of studies included in this meta-analysis.

Study and group	Median age	Induction regimen	Maintenance regimen	Number of enrolled	Male (%)	Stage of disease
J. Patel (2013) [11]						

Intervention arm	65	(Pem)500mg/m2, (C)AUC6, (Bev)15mg/kg four 21-day cycles	(Pem)500 mg/m2, (Bev) 15 mg/kg	472	251(53.2%)	IIIB~IV
Control arm	65	(Pac) 200 mg/m2, (C)AUC6, (Bev) 15mg/kg four 21-day cycles	(Bev) 15 mg/kg	467	249(53.3%)	

F. Barlesi (2013) [12]						

Intervention arm	60	(Pem)500mg/m2 (Bev)7.5 mg/kg, (Cis)75mg/m2 four 21-day cycles	(Pem)500mg/m2 (Bev)7.5 mg/kg,	128	72(57.6%)	IIIB~IV
Control arm	60	(Pem)500mg/m2 (Bev)7.5 mg/kg, (Cis)75mg/m2 four 21-day cycles	(Bev)7.5 mg/kg	125	68(56.7%)	

M. Karayama (2016) [13]						

Intervention arm	65	(Pem)500mg/m2, (C)AUC6, (Bev)15mg/kg four 21-day cycles	(Pem)500 mg/m2, (Bev) 15 mg/kg	55	35(63.6%)	IIIB~IV
Control arm	66	(Pem)500mg/m2, (C)AUC6, (Bev)15mg/kg four 21-day cycles	(Pem)500 mg/m2	55	39(70.9%)	

Pem, pemetrexed; Bev, bevacizumab; C, carboplatin; Pac, paclitaxel; Cis, cisplatin; AUC, area under the serum concentration-time curve

**Table 2:** Evaluation of risk of bias in included studies.

Study	Random sequence generation	Concealment of allocation	Blinding	Incomplete outcome (attrition bias)^*∗*^	Selective reporting	Other biases
J. Patel (2013) [11]	Yes	Not informed	No	I: 30/472 C: 24/467	No	No

F. Barlesi (2013) [12]	Yes	Not informed	No	I: 3/128 C: 5/125	No	No

M. Karayama (2016) [13]	Yes	Yes	No	I: 10/55 C: 20/55	No	No

^*∗*^Number of participants missing after randomization. I, intervention arm; C, control arm

**Table 3:** Meta-analysis of grade 3-4 adverse events.

Adverse event	Events in intervention arm	Events in control arm	RR (95% CI)	*P*
Neutropenia	93/462	139/453	0.66(0.52-0.82)	0.000
Thrombocytopenia	71/462	13/453	5.36(3.01-9.53)	0.000
Anemia	53/462	5/453	10.39(4.19-25.76)	0.000
Febrile neutropenia	7/462	14/453	0.49(0.20-1.20)	0.120
Fatigue	39/462	11/453	3.48(1.80-6.70)	0.000
Hypertension	24/462	30/453	0.78(0.47-1.32)	0.361
Overall	340/462	250/453	1.59(1.07-2.36)	0.022

## References

[1] Jemal A., Center M. M., DeSantis C., Ward E. M. (2010). Global patterns of cancer incidence and mortality rates and trends. *Cancer Epidemiology, Biomarkers & Prevention*.

[2] SEER Cancer Statistics Factsheets: Lung and Bronchus Cancer. National Cancer Institute. Surveillance, Epidemiology, and End Results Program. http://seer.cancer.gov/statfacts/html/lungb.html.

[3] National Comprehensive Cancer Network. Non–small cell lung cancer (version 1.2018). http://www.nccn.org/professionals/physician_gls/.

[4] Park J. O., Kim S. W., Ahn J. S., et al. (2007). Phase III trial of two versus four additional cycles in patients who are nonprogressive after two cycles of platinum-based chemotherapy in non-small-cell lung cancer. *Journal of Clinical Oncology*.

[5] Hotta K., Matsuo K., Ueoka H., Kiura K., Tabata M., Tanimoto M. (2004). Meta-analysis of randomized clinical trials comparing cisplatin to carboplatin in patients with advanced non-small-cell lung cancer. *Journal of Clinical Oncology*.

[6] Schiller J. H., Harrington D., Belani C. P. (2002). Comparison of four chemotherapy regimens for advanced non-small-cell lung cancer. *The New England Journal of Medicine*.

[7] Ciuleanu T., Brodowicz T., Zielinski C. (2009). Maintenance pemetrexed plus best supportive care versus placebo plus best supportive care for non-small-cell lung cancer: a randomised, double-blind, phase 3 study. *The Lancet*.

[8] Fidias P. M., Dakhil S. R., Lyss A. P. (2009). Phase III study of immediate compared with delayed docetaxel after front-line therapy with gemcitabine plus carboplatin in advanced non-small-cell lung cancer. *Journal of Clinical Oncology*.

[9] Sandler A., Gray R., Perry M. C. (2006). Paclitaxel-carboplatin alone or with bevacizumab for non-small-cell lung cancer. *The New England Journal of Medicine*.

[10] Paz-Ares L., de Marinis F., Dediu M. (2012). Maintenance therapy with pemetrexed plus best supportive care versus placebo plus best supportive care after induction therapy with pemetrexed plus cisplatin for advanced non-squamous non-small-cell lung cancer (PARAMOUNT): a double-blind, phase 3, randomised controlled trial. *The Lancet Oncology*.

[11] Patel J. D., Socinski M. A., Garon E. B. (2013). PointBreak: A randomized phase III study of pemetrexed plus carboplatin and bevacizumab followed by maintenance pemetrexed and bevacizumab versus paclitaxel plus carboplatin and bevacizumab followed by maintenance bevacizumab in patients with stage IIIB or IV nonsquamous non-small-cell lung cancer. *Journal of Clinical Oncology*.

[12] Barlesi F., Scherpereel A., Rittmeyer A. (2013). Randomized phase III trial of maintenance bevacizumab with or without pemetrexed after first-line induction with bevacizumab, cisplatin, and pemetrexed in advanced nonsquamous non-small-cell lung cancer: AVAPERL (MO22089). *Journal of Clinical Oncology*.

[13] Karayama M., Inui N., Fujisawa T. (2016). Maintenance therapy with pemetrexed and bevacizumab versus pemetrexed monotherapy after induction therapy with carboplatin, pemetrexed, and bevacizumab in patients with advanced non-squamous non small cell lung cancer. *European Journal of Cancer*.

[14] Gerber D. E., Schiller J. H. (2013). Maintenance chemotherapy for advanced non-small-cell lung cancer: new life for an old idea. *Journal of Clinical Oncology*.

[15] Moher D., Liberati A., Tetzlaff J., Altman D. G., The PRISMA Group (2009). Preferred reporting items for systematic reviews and meta-analyses: the PRISMA statement. *Annals of Internal Medicine*.

[16] Parmar M. K. B., Torri V., Stewart L. (1998). Extracting summary statistics to perform meta-analyses of the published literature for survival endpoints. *Statistics in Medicine*.

[17] Higgins J. P. T., Thompson S. G. (2002). Quantifying heterogeneity in a meta-analysis. *Statistics in Medicine*.

[18] Guyatt G. H., Oxman A. D., Vist G. E., et al. (2008). Rating quality of evidence and strength of recommendations: Going from evidence to recommendations. *British Medical Journal*.

[19] Pirker R., Pereira J. R., Szczesna A. (2009). Cetuximab plus chemotherapy in patients with advanced non-small-cell lung cancer (FLEX): an open-label randomised phase III trial. *The Lancet*.

[20] Brodowicz T., Krzakowski M., Zwitter M. (2006). Cisplatin and gemcitabine first-line chemotherapy followed by maintenance gemcitabine or best supportive care in advanced non-small cell lung cancer: A phase III trial. *Lung Cancer*.

[21] Pérol M., Chouaid C., Pérol D. (2012). Randomized, phase III study of gemcitabine or erlotinib maintenance therapy versus observation,with predefined second-line treatment, after cisplatin-gemcitabine induction chemotherapy in advanced non-small-cell lung cancer. *Journal of Clinical Oncology*.

[22] Gentzler R. D., Johnson M. L. (2015). Complex decisions for first-line and maintenance treatment of advanced wild-type non-small cell lung cancer. *The Oncologist*.

[23] Patel J. D., Hensing T. A., Rademaker A. (2009). Phase II study of pemetrexed and carboplatin plus bevacizumab with maintenance pemetrexed and bevacizumab as first-line therapy for nonsquamous non-small-cell lung cancer. *Journal of Clinical Oncology*.

[24] Dahlberg S. E., Ramalingam S. S., Belani C. P., Schiller J. H. (2011). A randomized phase III study of maintenance therapy with bevacizumab (B), pemetrexed (Pm), or a combination of bevacizumab and pemetrexed (BPm) following carboplatin, paclitaxel and bevacizumab (PCB) for advanced nonsquamous NSCLC: ECOG trial 5508 (NCT01107626). *Journal of Clinical Oncology*.

[25] Gentzler R. D., Patel J. D. (2014). Optimal first-line and maintenance treatments for advanced-stage nonsquamous non-small cell lung cancer. *JNCCN — Journal of the National Comprehensive Cancer Network*.

[26] Johnson B. E., Kabbinavar F., Fehrenbacher L. (2013). ATLAS: Randomized, double-blind, placebo-controlled, phase IIIB trial comparing bevacizumab therapy with or without erlotinib, after completion of chemotherapy, with bevacizumab for first-line treatment of advanced non-small-cell lung cancer. *Journal of Clinical Oncology*.

[27] Zinner R. G., Obasaju C. K., Spigel D. R. (2015). PRONOUNCE: Randomized, open-label, phase III study of first-line pemetrexed + carboplatin followed by maintenance pemetrexed versus paclitaxel + carboplatin + bevacizumab followed by maintenance bevacizumab in patients with advanced nonsquamous non-small-cell lung cancer. *Journal of Thoracic Oncology*.

